# Cariprazine & clozapine: A systematic review of a promising combination in the management of treatment-resistant schizophrenia

**DOI:** 10.1192/j.eurpsy.2025.563

**Published:** 2025-08-26

**Authors:** S. Pappa, Z. B. Dombi, E. Caldwell-Dunn, R. Csehi, Á. Barabássy

**Affiliations:** 1 Imperial College London; 2 West London NHS Trust, London, United Kingdom; 3 Gedeon Richter Plc., Budapest, Hungary

## Abstract

**Introduction:**

Up to 30-70% of patients with treatment-resistant schizophrenia (TRS) remain symptomatic despite gold standard treatment, clozapine. To date, commonly used antipsychotics have demonstrated little therapeutic benefit as augmenting agents in comparison to placebo. Emerging evidence suggests that novel D2-D3 partial agonist cariprazine is a promising augmentation strategy to clozapine for TRS.

**Objectives:**

This systematic review aims to collect the available real-world evidence of effectiveness and tolerability of cariprazine and clozapine combination treatment.

**Methods:**

A systematic review was performed using PubMed, MEDLINE, EMBASE and Cochrane databases from January 2017 until September 2024 for cases where cariprazine was used as an augmentation strategy for clozapine with the following terms: (cariprazin*) AND (clozapin*) AND (‘case report*’ OR ‘case report’/de OR ‘case stud*’ OR ‘case study’/de OR ‘case seri*’ OR ‘add-on’ OR augmentation OR combin*).

**Results:**

After removal of duplicates, 108 studies were retrieved, of which 20 studies were included (one prospective pilot study and 19 case reports). Total cases comprised of 47 patients (30 male, 17 female), with diagnoses of schizophrenia (n=40), schizoaffective disorder (n=6) and emotionally unstable personality disorder (EUPD) and autism spectrum disorder (n=1). Patients were treated with clozapine (dose range 37.5-850 mg/day) and cariprazine (doses 1.5-6.0 mg/day) for a median of 122 days (range 18-456). Although a variety of subjective and objective outcome measures were employed, cariprazine was generally found to be a well-tolerated and effective adjunct to clozapine in a wide range of different symptom profiles; demonstrating efficacy across positive, negative and affective symptoms, quality of life and global functioning in the majority of cases. Additional benefits of weight loss and improving commonly experienced adverse side effects of clozapine were also frequently reported. In 3 cases cariprazine augmentation did not improve symptomatology, whereas in 6 cases the combination resulted in the exacerbation of different symptoms such as anxiety or restlessness.

**Conclusions:**

By targeting different receptors, cariprazine and clozapine appear to act synergistically allowing for a well-tolerated and effective antipsychotic combination. Large-scale RCTs are warranted to further evaluate its effectiveness compared to placebo.

**Disclosure of Interest:**

S. Pappa: None Declared, Z. Dombi Employee of: I am an employee of Gedeon Richter Plc., originator of cariprazine., E. Caldwell-Dunn: None Declared, R. Csehi Employee of: I am an employee of Gedeon Richter Plc., originator of cariprazine., Á. Barabássy Employee of: I am an employee of Gedeon Richter Plc., originator of cariprazine.

